# Supplementary material for: Neurological Surveillance in Moderate-Late Preterm Infants—Results from a Dutch–Canadian Survey
Source: Children (Basel). 2022 Jun 8;9(6):846. doi: 10.3390/children9060846 (PMC9221620; doi:10.3390/children9060846)
Supplement: Supplementary file 1 [file children-09-00846-s001.zip › children-1752333-supplementary.pdf]

Supplemental material – Questions BIMP survey

General Questions about responder:

1. In which country are you employed?
   a. The Netherlands
   b. Canada
2. Number of years working as a paediatrician (-neonatologist)?
   a. < 5 years
   b. 5 – 10 years
   c. 10 – 15 years
   d. > 15 years
3. Position
   a. Paediatrician
   b. Paediatrician-neonatologist
4. Hospital
   a. With level III NICU
   b. With level I-II neonatal unit
   c. Other
5. Number of (incubator) beds
   a. < 5
   b. 5-10
   c. 10-15
   d. 15-20
   e. > 20

Specific questions related to moderate and late preterm (MLPT) infants

A. Admission criteria

6. Are there guidelines for admission of MLPT infants to your unit?
   a. Yes
   b. No
7. If yes, are these criteria recorded in hospital guidelines

If yes to question 6→ please proceed to questions 8-16 about these criteria

8. Gestational age cut-off
   a. Yes (<….weeks) please specify cut-off
   b. No
9. Birthweight cut-off
   a. Yes (<…..gram); please specify cut-off
   b. No
10. Hypoglycaemia during postnatal day 1-3 (including day 3)
    a. Yes (<….mg/dl/ mmol/l); please specify cut-off
    b. No
11. Hyperbilirubinemia needing treatment
    a. Yes
    b. No
12. (Suspected) sepsis
    a. Yes
    b. No
13. Feeding difficulties (not drinking well, vomiting, delayed meconium passage)
    a. Yes
    b. No
14. Suboptimal start (Apgar score < 7 at 5 minutes)
    a. Yes
    b. No
15. Respiratory distress
    a. Yes
    b. No
16. Other guidelines
    a. Yes (please specify)
    b. No

17. Do you have any additional comments regarding questions 8-16?

B. Laboratory testing

18. Do you perform routine laboratory testing in admitted MLPT infants?
    a. Yes
    b. No

If yes, which tests are performed → Please proceed to questions 19-24

19. Glucose
    a. Yes
    b. No
    c. Only when indicated (clinical symptoms)
20. Bilirubin
    a. Yes
    b. No
    c. Only when indicated (clinical symptoms)
21. CRP (or other inflammatory parameter)
    a. Yes
    b. No
    c. Only when indicated (clinical symptoms)
22. Haemoglobin (and/or other haematological parameters)
    a. Yes
    b. No
23. Other
    a. Yes (please specify)
    b. No
24. Do you have any additional comments regarding questions 19-23?

C. Clinical condition of MLPT infant who is not admitted to neonatal unit

25. Is the clinical condition of MLPT infants monitored if they are not admitted to the neonatal unit?

If yes → please proceed to questions 26-31

26. MLPT neonates are staying on the maternity ward
    a. On first postnatal day
    b. Until postnatal day 2
    c. Until postnatal day 3
    d. Other (please specify)
27. Through telephone contact with parents
    a. Yes; if yes: when and by whom?
    b. No
28. Through contact with midwife and/or maternity/community nurse
    a. Yes
    b. No
29. Through out-patient check-up during the first week
    a. Paediatrician
    b. Family doctor
    c. Well-baby clinic
    d. No
30. Through routine laboratory tests
    a. Yes (please specify)
    b. No
31. Do you have any additional comments regarding questions 26-31?

D. Screening the MLPT brain

32. Does your unit have a guideline for screening of the brain of MLPT infants?
    a. Yes

b. No

33. If yes, are these guidelines generally followed?
    a. Yes
    b. No
34. If not, please specify why these guidelines are not followed?

35. Do MLPT infants routinely undergo one or more cranial ultrasound (cUS) examinations?
    a. Yes; please proceed to questions 36-35
    b. Only when indicated; please proceed to questions 40-52
    c. No; please proceed to question 53

<u>Questions regarding routine cUS (36-39)</u>

36. At what postnatal age is first cUS done?
    a. 1-3 days
    b. 4-7 days
    c. > 7 days
    d. Around term equivalent age / prior to discharge
37. How frequently is cUS done during hospital admission?
    a. 1x
    b. 2x
    c. =/> 3x
38. Is there a gestational age cut-off to do cUS examinations? Only when:
    a. < 33 weeks
    b. < 34 weeks
    c. < 35 weeks
    d. < 36 weeks
    e. No
39. Who performs the cUS examinations in MLPT infants? Tick all that apply
    a. Radiologist or radiology technician
    b. Paediatrician
    c. (fellow)Neonatologist
    d. (paediatric)neurologist
    e. Nurse practitioner/ physician assistant/nurse specialist

<u>Questions regarding specific indications cUS (40-52)</u>
<u>Do you preform cUS in case of the following conditions:</u>

40. (Suspected) Seizures
    a. Yes
    b. No
41. Other neurological symptoms (such as jitteriness, irritability, excessive crying, abnormal muscle tone, lethargy)
    a. Yes (please specify)
    b. No
42. Suspected sepsis
    a. Yes
    b. No
43. Confirmed sepsis
    a. Yes
    b. no
44. Suspected meningitis
    a. Yes
    b. No
45. Confirmed meningitis
    a. Yes
    b. no
46. Anaemia, needing RBC transfusions
    a. Yes, please specify cut-off
    b. No
47. Hyperbilirubinemia needing exchange transfusion

a. Yes
b. No

48. Antenatal diagnosis or suspicion of brain anomaly
    a. Yes
    b. No
49. Dysmorphia
    a. Yes
    b. No
50. Other
    a. Yes (please specify)
    b. No
51. Are these indications (40-50) adhered to the same by all your colleagues?
    a. Yes
    b. No
52. If no, please specify?

53. Do MLPT infants routinely undergo MRI?
    a. Yes; please proceed to questions 54-55
    b. No; please proceed to question 56
    c. Only when indicated;

<u>Questions regarding brain MRI</u>

54. Is there experience with doing MRI in MLPT infants in your unit?
    a. Yes (> 10 infants/year)
    b. Limited (1-10 infants/year)
    c. No, the infant is referred to a level III hospital for MRI
55. If yes, are there specific indications for neonatal MRI?
    a. Yes; please specify
    b. No

<u>Questions regarding neurological examinations and follow-up programs</u>

56. Do you perform routine neurological examinations in MLPT infants?
    a. Yes
    b. No
57. If yes, when is this performed?
    a. 1st postnatal day
    b. Before discharge
    c. Other; please specify
58. Do MLPT infants enroll in a standard follow-up program in your hospital?
    a. Yes
    b. No
    c. If indicated
59. If yes to question 58, what are the indications (please tick all that apply)?
    a. Gestational age < 35 weeks
    b. Gestational age < 34 weeks
    c. Gestational age < 33 weeks
    d. Low birthweight
    e. Other (please specify)
60. If answer a. or c. to question 58, who performs the follow-up care?
    a. Paediatric nurse
    b. Paediatrician
    c. Paediatric resident
    d. Nurse practitioner/ physician assistant/nurse specialist
    e. Other (please specify)
61. If answer a. or c. to question 58 the follow-up consists of (please tick all that apply):
    a. Weight, length and head circumference measurements
    b. Answering parents questions
    c. Physical examination

    d. Neurological examination
    e. Nutritional advice
    f. Developmental assessment
    g. Other (please specify)

If answer a. or c. to question 58, is there a collaboration with other disciplines or services?

62. Physiotherapist
    a. Yes
    b. No
63. Other services (i.e. speech therapist, psychologist)
    a. Yes (please specify)
    b. No
64. Are MLPT infants seen together with another discipline?
    a. Yes
    b. No
65. If yes to question 64, please specify which discipline
    a. Physiotherapist
    b. General practitioner
    c. Well baby doctor or "jeugdarts" (the Netherlands)
66. Until what age or milestone is follow-up performed in MLPT infants?
    a. 1 month
    b. 2-3 months
    c. 3-12 months
    d. When the child has started walking
    e. Other (please specify)

Concluding questions

67. Do you feel that current clinical practice/care on neuroimaging and neurological development in MLPT infants during admission to your unit is satisfactory?
    a. Very satisfied
    b. Reasonably satisfied
    c. Neutral
    d. Unsatisfied
68. If you are not satisfied, how would you like to improve this?
69. Do you feel that current clinical practice/care on neuroimaging and neurological development in MLPT infants after discharge is satisfactory?
    a. Satisfactory
    b. Reasonably satisfactory
    c. Neutral
    d. Unsatisfactory
70. If you are not satisfied, how would you want to improve this?
71. If you have any final comments, please note them here

This is the end of the survey. We would like to thank you for your cooperation and time.
END of the survey
